# Uncomplicated Wide Oblique Clivus Fracture, the First Case to be Included in the List of Classification; Report of the Case and Review of Literature

**DOI:** 10.29252/beat-070412

**Published:** 2019-10

**Authors:** Sina Jelodar, Ahmad Pourrashidi, Abbas Amirjamshidi

**Affiliations:** 1 *Department of Neurosurgery, Sina Hospital, Emam Ave. Tehran University of Medical Sciences, Tehran, Iran*

**Keywords:** Clivus fracture, Skull base fracture, Traumatic brain injury

## Abstract

Sheno-occipital diastasis happens more frequently in children and is accompanied with neural and vascular injuries leading to a high rate of mortality. We present a rare type of clival fracture in a 21 years old man who could survive without any deficit even though the fracture extended widely from left Asterion to the right orbit accompanied by widespread damage of the skull base air sinuses in 3D CT scan. To the best knowledge of the authors, neither this type of fracture, nor the clinical presentation, has been reported in relevant literature.

## Introduction

We present a 20-year-old boy with pneumocephalus after direct blow to the left occiput and review the literature briefly. Clivus is an uncommon site for skull base fracture. Population based studies show that clivus fracture occurs most commonly in pedestrians involved in traffic accidents (Table 1). The extent and type of fracture depends on the trajectory and magnitude of the trauma [1-8]. Longitudinal and oblique fractures are suggested as the more devastating types in different studies [2]. There was an oblique clivus fracture (CVF) with sphenooccipital dehiscence but the patient had no neurological deficit. We would like to suggest this type of skull base fracture to be included in the classification skull base fractures.

## Case Report

**Fig. 1:**
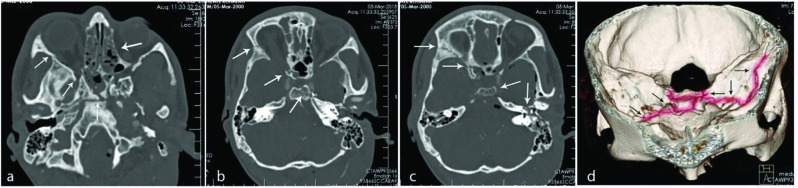
White arrow in the middle showing the horizontal and vertical fracture lines and dehiscence of the clivus with axial subluxation to the left (a). Fracture line is demarcated using white arrow starting from the base of the right mastoid bone coming to the lateral and mid clivus (b). Arrows showing fractured left anterior clinoid process in figures (c). Extension of the fracture line to the left orbital plate and sphenoid wing (a, b). 3D reconstruction of the skull base region shows whole the fracture line shaded to highlight the CVF all along the skull base (d).

A 20-year-old man was brought to our level 1 trauma center with severe headache after a direct blow to the head on the left side of the occiput. He was conscious, oriented and had a good memory of the moment of the injury. We found a superficial laceration of the scalp on the left occiput and no rhinorrhea or ottrrhagia. The neurological examination was completely normal, and the patient had fluent, clear speech. A head CT scan revealed diffuse pneumocephalous with the pattern of the gyri. Paranasal sinuses were filled with cerebro-spinal fluid (Figure 1). There was a linear skull base fracture, extending from the left petrous bone, crossing the sphenooccipital synchondrosis and extending to the roof of orbit on the right side (Figure 1). The fracture line traversed the sphenoid sinus, the right anterior clinoid process and the right sphenoid wing also. No insult to the internal carotid artery (ICA) or vertebral and basilar arteries were detected in CTV. Patient was admitted to the intensive care unit and treated with complete bed rest and continuous high concentration oxygen mask. The pneumocephalus decreased remarkably and patient was transferred to the ward after three days. He was discharged home after seven days and was asymptomatic when last visited after 3 months. Appropriate informed consent is taken from the patient and is present in the file.

## Discussion

Clivus is a thick part of the occipital bone that forms a gradual sloping process at the anterior most portion of the basilar occipital bone at its junction with the sphenoid bone via ‘spheno-occipital synchondrosis’ and the petrosal part of the temporal bone on each side [1]. The cartilaginous connection between the clivus and the body of sphenoid is ossified in the adolescence up to the age of 15 and skull is more prone for clivus dehiscence in childhood [5,6]. Our case was a 20-year-old man.

**Table 1:** Showing the list of other cases of skull base fracture with sufficient information to be compared to our case

	**Age/ ** **gender **	**Cranial fracture **	**Neurologic deficit, additional findings **	**Prognosis **
Khan and Zumstein	19/M	Fracture in petrous bone	Deficit in cranial nerves 3, 6 and 7	GCS: 15
Arizavakan et al.	18/M	Left anterior cranial fossa base Fracture in roof of right orbit	Ophthalmoplegia and bilateral multiple cranial nerve palsies Diffuse pneumocephalus with extension into lateral Subarachnoid hemorrhage	–
Evers et al.	43/M	Left occipital fracture Fracture of the atlas, anterior arch lower end	Retrograde amnesia Mild motor deficit	Home
Okten et al.	19/M	Fracture in petrous bone	GCS: 3-4	Death
Ochalski et al.	32/F	Petro-occipital fracture Occipitomastoid fracture	SDH	GCS: 5, home
	16/M	–	SAH ICA dissection	Rehabilitation
	15/F	Sphenooccipital fracture Petrooccipital fracture Occipitomastoidal fracture	EDH, SDH, contusion, Bilat MCA & ACA infarcts	Brain death
	6/M	Occipital hematoma and fracture	SAH, EDH CN VII palsy, Bilat CN VIII palsy	Home
	16/M	–	SAH ICA dissection GCS: 7	Rehabilitasyon
Menkü et al.	25 M	Transverse	Right CN VI palsy	Improvement
	17 M	Transverse	Left CN III palsy Bilateral CN VI palsy	Death, 7 days
	45 F	Transverse	CN II palsy Bilateral CN VI palsy Left CN VII palsy Left Blindness	
	54 M	transverse	Left VI	Improvement
	68 M	Longitudinal	Rt III VII	Death
	18 M	Longitudinal	Lt VII	Lt hemiparesis
	32 M	longitudinal	Lt III, bilat VI, Lt VII	Lt VII
	48 F	Longitudinal	Lt VII	Death
	38 F	Longitudinal	Right III, VI, VII	Death
Sanders and Vander	16/F	transverse	CN V, VI, VII paralysis Horner syndrome	GCS: 15
	26	transverse	CN VI,VII paralysis, Horner syndrome	GCS: 15
	9/F	transverse	CN III, IV, V, VI,VII paralysis Horner syndrome ICA stenosis	GCS: 15
Kapila and Chakeres	21M	Transvers cliv fracture	Bilateral CN VI paralysis CSF otorrhea CCF	GCS: 15
Corradino et al. Joslyn et al.	32 y	Transvers clivus fracture	CN III, IV, V paralysis Bilateral CN VI, VII paralysis	Death
Akar, Ö et al.	38/M	Small sxial fracture lowest part of clivus	no deficit	GCS: 15, home
Our case	20 M	Fracture from Rt. Asterion to Let orbit with clival dehescenc	No deficit	GCS: 15, home

Fracture of the clivus is a rare event and is seen only in 0.21% - 0.56% of traumatic head injuries. The trauma is usually a blunt one with compressive nature. The compressive pattern of the trajectory of the trauma or vector of the force is of main importance in development of CVF in most of the reported cases [4]. In our patient the fracture occurred due to a direct blunt trauma to the left Asterion. The vector of the force created an oblique line of fracture that extended from the left petrous bone to the sphenooccipital synchondrosis, then to the right anterior clinoid process and crossed to the roof of the right orbit (Figures 1A, B, C, D). The sphenoid and the ethmoid sinuses were also smashed by the vector of force passing through the skull base in this case. 

 Corradino *et al*., [2] 1990, classified clival fractures to longitudinal, horizontal and oblique shapes. Wrinckler-Schwarts A *et al*., [8] subdivided the fracture types according to the extent of bonny damage. Neither of the authors’ classifications nor the existing literature can describe the CVFs in a convincing way for each case. That is one of the main reasons to present this case of ours with diastatic/displaced CVF as a very rare event and as a sample to be included in further multicentral studies for classification of CVFs.

The reported morbidity and mortality rate have been between 24% and 31% in different studies [3,4]. One of the theories to explain the death at the scene of traumatic brain injuries (TBI) after clivus fracture is that, the basilar artery may be crushed by the separated bone fragments [1]. Direct or indirect trauma to the cranial nerves can explain the neurological deficits usually occurring in such cases. In our case, a unilateral blunt trauma which was not severe enough to cause brain parenchymal damage, lead into this rare type of sphenooccipital dehiscence without any morbidity. We highlighted the main take away message of this case which can be important for neuro-trauma surgeons and radiologists.

In conclusion, a rare case of CVF is presented to be enrolled in the classification list of the clival fractures. 

## Declarations:

The ethics committee of Sina Hospital approved resenting the data of the file of the patient in this report.

No grant, financial support or funding for any of the authors.

Appropriate informed consent is taken from the patient and is present in the file.

All the authors contributed in preparation of the manuscript equally.

## Conflict of Interest:

The authors declare that they have no competing interests.
